# Case report: Equine metacarpophalangeal joint partial and full thickness defects treated with allogenic equine synovial membrane mesenchymal stem/stromal cell combined with umbilical cord mesenchymal stem/stromal cell conditioned medium

**DOI:** 10.3389/fvets.2024.1403174

**Published:** 2024-05-22

**Authors:** I. L. Reis, B. Lopes, P. Sousa, A. C. Sousa, A. Rêma, A. R. Caseiro, I. Briote, A. M. Rocha, J. P. Pereira, C. M. Mendonça, J. M. Santos, L. Lamas, L. M. Atayde, R. D. Alvites, A. C. Maurício

**Affiliations:** ^1^Departamento de Clínicas Veterinárias, Instituto de Ciências Biomédicas de Abel Salazar (ICBAS), Universidade do Porto (UP), Porto, Portugal; ^2^Centro de Estudos de Ciência Animal (CECA), Instituto de Ciências, Tecnologias e Agroambiente da Universidade do Porto (ICETA), Porto, Portugal; ^3^Associate Laboratory for Animal and Veterinary Science (AL4AnimalS), Lisboa, Portugal; ^4^Cooperativa de Ensino Superior Politécnico e Universitário (CESPU), Avenida Central de Gandra, Gandra, Portugal; ^5^Departamento de Ciências Veterinárias, Escola Universitária Vasco da Gama (EUVG), Coimbra, Portugal; ^6^Centro de Investigação Vasco da Gama (CIVG), Escola Universitária Vasco da Gama (EUVG), Avenida José R. Sousa Fernandes, Coimbra, Portugal; ^7^Campus Agrário de Vairão, Centro Clínico de Equinos de Vairão (CCEV), Vairão, Portugal; ^8^Faculdade de Medicina Veterinária, Universidade de Lisboa, Lisboa, Portugal; ^9^CIISA—Centro Interdisciplinar-Investigação em Saúde Animal, Faculdade de Medicina Veterinária, Av. Universidade Técnica de Lisboa, Lisboa, Portugal

**Keywords:** case report, equine, osteochondral defect, synovial membrane mesenchymal stromal/stem cell, umbilical cord conditioned medium, cell-based medicinal product

## Abstract

Here, we describe a case of a 5-year-old show-jumping stallion presented with severe lameness, swelling, and pain on palpation of the left metacarpophalangeal joint (MCj). Diagnostic imaging revealed full and partial-thickness articular defects over the lateral condyle of the third metacarpus (MC3) and the dorsolateral aspect of the first phalanx (P1). After the lesion’s arthroscopic curettage, the patient was subjected to an innovative regenerative treatment consisting of two intra-articular injections of equine synovial membrane mesenchymal stem/stromal cells (eSM-MSCs) combined with umbilical cord mesenchymal stem/stromal cells conditioned medium (UC-MSC CM), 15 days apart. A 12-week rehabilitation program was accomplished, and lameness, pain, and joint effusion were remarkably reduced; however, magnetic resonance imaging (MRI) and computed tomography (CT) scan presented incomplete healing of the MC3’s lesion, prompting a second round of treatment. Subsequently, the horse achieved clinical soundness and returned to a higher level of athletic performance, and imaging exams revealed the absence of lesions at P1, fulfillment of the osteochondral lesion, and cartilage-like tissue formation at MC3’s lesion site. The positive outcomes suggest the effectiveness of this combination for treating full and partial cartilage defects in horses. Multipotent mesenchymal stem/stromal cells (MSCs) and their bioactive factors compose a novel therapeutic approach for tissue regeneration and organ function restoration with anti-inflammatory and pro-regenerative impact through paracrine mechanisms.

## 1 Introduction

Human and animal athletes are prone to articular lesions due to trauma or stress-induced pathologies. The high-impact nature of equine sports leaves their articular cartilage particularly susceptible to damage (1). Articular defects might be partial-thickness, affecting only articular cartilage, or full-thickness, also reaching subchondral bone. Full-thickness defects may occur in young and mature sport horses, secondary to stress-related trauma when cartilage is exposed to excessive loading forces (2).

Cartilage tissue has low regenerative efficiency due to its avascular nature, among other factors (3). This poor intrinsic ability to heal favors the development of osteoarthritis (OA), a common chronic joint disease characterized by pain, deformity, instability, and reduction of motion and function (4). OA’s pathologic findings might include articular cartilage degradation associated or not with subchondral bone thickening, osteophyte formation, synovial inflammation, ligament degeneration, and capsule hypertrophy (5). Traditional treatments predominantly focus on pain and inflammation control, relieving symptoms but having limited ability to correct the underlying pathology, to delay disease progress and to, ultimately, regenerate damaged tissues (6). When conservative treatment fails, surgical approaches, such as autologous chondrocyte implantation (ACI), might be considered but present limitations: they do not alter disease progression, do not regenerate cartilage, and do not enhance organ function (7). They usually result in inferior quality cartilage (fibrocartilage) formation, donor site morbidity, loss of phenotype from differentiation of primary chondrocytes during expansion, and possible need for open surgery. These techniques are also limited by low tissue availability (8). Hence, there is a pressing demand for novel treatments capable of regenerating tissues and restoring functionality, enabling horses to resume physical activities and athletic performance (1, 4, 7, 9).

Dedicated research in regenerative medicine has led to the creation of novel therapeutics targeting cartilage and bone pathologies. These advancements focus on modulating and inhibiting disease progression while promoting tissue regeneration, ultimately aiming for a return to anatomical and physiological function closely resembling the original tissue properties (10, 11). The overarching goal is to shift the joint’s state from an inflammatory and catabolic nature to an anabolic state, enabling cartilage regeneration.

Implantation of MSCs into cartilage defects has shown great promise in both cartilage and subchondral bone repair (12–16) as they release bioactive factors, promoting repair and regeneration. There is evidence that synovial membrane MSCs (SM-MSCs) present a greater chondrogenic ability among MSCs from other origins, suggesting their superiority in cartilage repair (17–23). These cells have close anatomical contact with cartilage, suggesting a close bias toward the production of cartilage, becoming a good candidate for cartilage tissue engineering (19).

Research also supports the use of umbilical cord MSCs (UC-MSCs) for cartilage regeneration (24, 25). UC-MSCs from Wharton’s jelly have high potential for proliferation, differentiation, and lower immunogenicity (12). UC-MSCs can stimulate *in vitro* production of key cartilage components, such as glycosaminoglycans and collagen type II (24). In addition, MSC-conditioned medium (MSC-CM) is emerging as a promising therapy for cartilage repair as a cell-free therapy, presenting all of the advantages of the bioactive factors secreted by MSCs, such as cytokines, chemokines, proteins, extracellular vesicles, and growth factors. These components exhibit both anti-inflammatory and pro-inflammatory effects, influencing chondrocyte processes and enhancing cartilage structure and biomechanical properties (26–30). By modifying the local inflammatory environment, CM may slow down OA progression by reducing harmful metabolites for articular cartilage ECM and chondrocytes produced by activated cells in the joint (27, 31).

In consideration of the aforementioned parameters, a therapeutic product has been developed, combining eSM-MSC with UC-MSC CM. The use of eSM-MSC in treating equine tendonitis and desmitis has already been demonstrated to have very good and promising results in tissue regeneration (32). Additionally, the combined use of eSM-MSC and UC-MSC CM has been previously employed and described in the treatment of a long medial collateral ligament of an equine tarsus, also presenting very favorable results in ligament regeneration, absence of clinical signs, and return to sportive performance and competition (33). The present study introduces the application of this combination in addressing two distinct articular defects: full-thickness and partial-thickness, and intends to understand and describe its regenerative ability.

## 2 Case description

### 2.1 Clinical description

A 5-year-old showjumper stallion was evaluated due to an acute lesion of the left forelimb (LF) metacarpophalangeal (MCj) with associated lameness.

**Figure 1:**
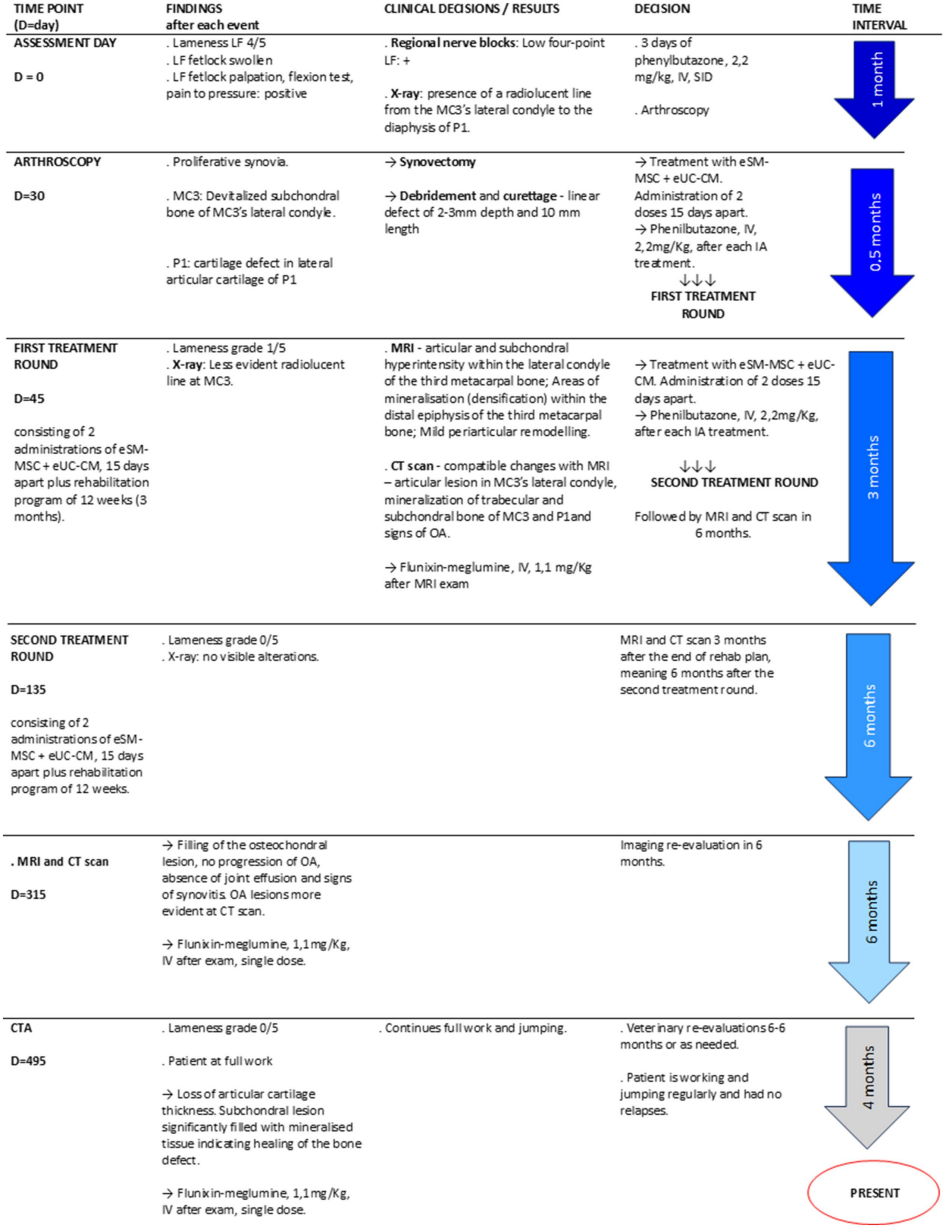
Treatment timeline.

On the assessment day, the MCj was swollen, and the horse presented a lameness grade of 4/5 and scored accordingly with the AAEP lameness grading scale (34). Palpation, manipulation, flexion test, and pain to pressure were performed, and all had a positive reaction (Figure 1, Day = 0).

As complementary diagnostic exams, regional nerve blocks, as well as radiographs (X-rays), arthroscopy, magnetic resonance image (MRI), computer tomography (CT), and computed tomography arthrography (CTA), were performed at different times of the therapeutical intervention.

The patient did not receive any other medical treatments for at least 2 months before and after the cell-based treatment (including non-steroidal anti-inflammatory drugs, intra-articular corticosteroids, hyaluronan, glycosaminoglycans, hemoderivative treatments, and other MSCs preparations), except for phenylbutazone (Phenylarthrite^®^, 2,2 mg/kg, IV, Vetoquinol, France), SID for 3 days on the assessment day, a single dose of phenylbutazone after every intra-articular (IA) treatment, and a single dose of flunixin-meglumine (Flunixin 3E^®^,1.1 mg/kg, IV, Norbrook Laboratories, Ireland) at MRI and CTA examinations.

Figure 1 presents a schematic timeline of the clinical case.

## 3 Diagnostic assessment

### 3.1 Regional nerve blocks

Three regional nerve blocks were performed on the LF: digital palmar, abaxial, and low four-point nerve block. Lidocaine 2% (Anestesin^®^ 20 mg/mL, Orion Corporation, Finland) was injected medially and laterally (2 mL per injection site) in each assessment with 25G needles. These localized the pain to the fetlock region (Figure 1, D = 0).

### 3.2 Diagnostic imaging

#### 3.2.1 Radiographs

Radiological examination (X-ray) of the LF MCj was performed with a digital system—CareRay Cw series^®^ (CareRay, Suzhou, China), radiological constants: 72 Kv, 0.8 mA. The distance between the X-ray generator (Orange 1,060 HF, EcoRay, Seoul, Republic of Korea) and the flat panel was approximately 66 cm. Seven standard views of the MCj were obtained, namely lateromedial (LM), dorsopalmar (DP), Latero-medial flexed (LM Flex), oblique dorsolateral-palmaromedial (DLPMO), oblique dorsomedial-palmarolateral (DMPLO), oblique palmarolateral-dorsomedial oblique (PLDMO), and Palmaromedial-Dorsolateral oblique (PMDLO). The radiological examination was performed on assessment day (Figure 1, Day = 0), and those views that presented alterations were repeated during the rehabilitation program, as described in Table 1.

**Table 1:** Physical rehabilitation program.

Week	Exercise
0–2	2 days: stall confinement Handwalk: 10 min Day 15: new treatment
3–4	2 days: stall confinement Handwalk: 10 min VET-CHECK + X-ray
5	Handwalk: 15 min
6	Handwalk: 20 min VET-CHECK
7	Handwalk: 25 min
8	Handwalk: 30 min VET-CHECK + X-ray
9–10	Handwalk: 30 min + 5 min trot
11–12	Handwalk: 30 min + 10 min trot VET-CHECK + MRI + CT scan (first treatment round)

**Figure 2:**
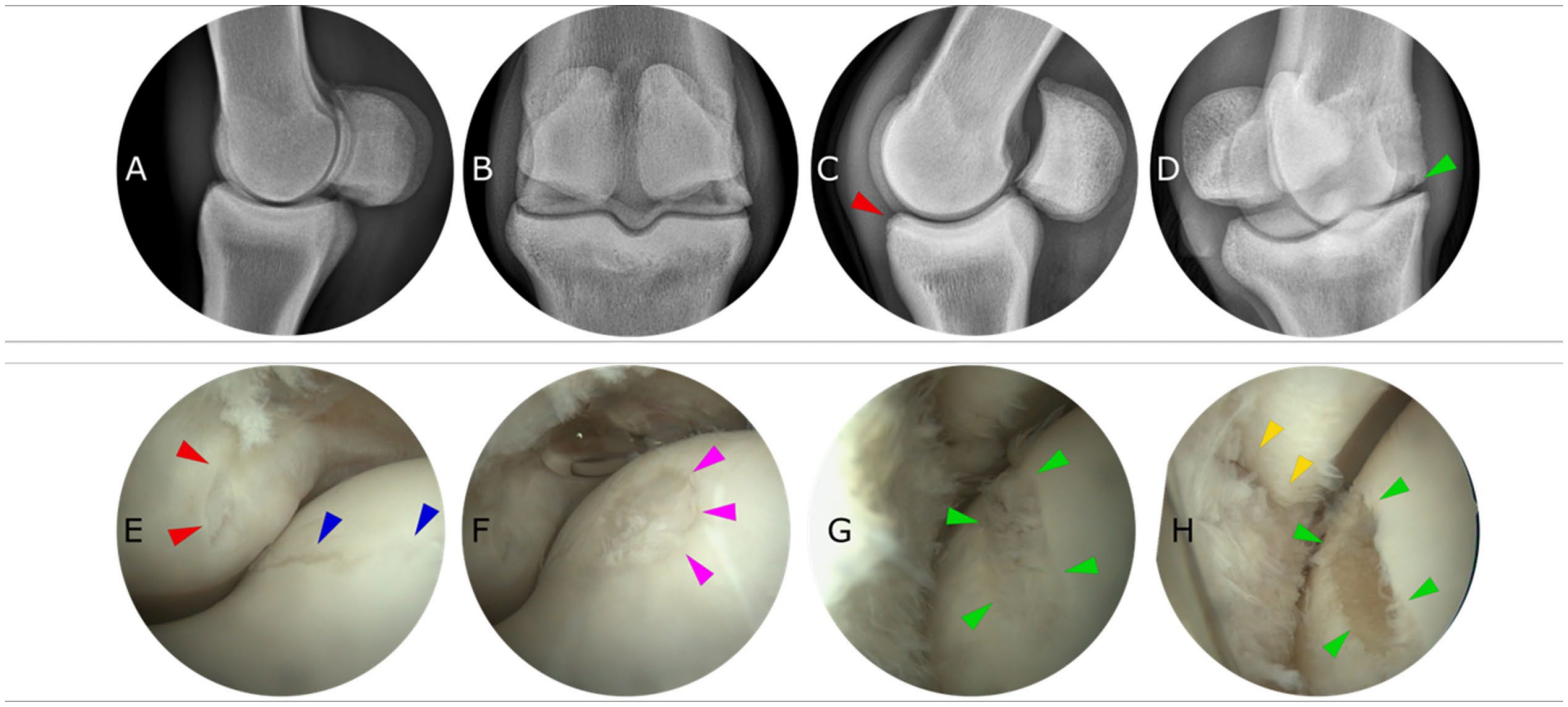
Pre-treatment diagnostic imaging and surgery: Radiographs and arthroscopy. Four radiographic projections **(A)** Lateromedial (LM), **(B)** Dorsopalmar (DP), **(C)** LM flexed, and **(D)** DMPLO (dorsomedialpalmarolateral Oblique) are presented. The slight radiological alterations are highlighted with a red (first phalanx) and green (MC3) arrow. Arthroscopic images of the LF metacarpophalangeal joint. **(E)** Red arrows present a semilunar partial thickness lesion on the dorsoproximal of P1 and blue arrows delimit a partial thickness liner lesion on the lateral aspect of the sagittal ridge of MC3. **(F)** Pink arrows present a circular partial thickness defect further proximal to the lateral aspect of the sagittal ridge of MC3. **(G,H)** show the main lesions on the dorso-distal aspect of the lateral condyle of MC3 (green arrows) before and after debridement of the lesion. Yellow arrows indicate an adjacent lesion on the dorsoproximal aspect of P1 (note that the joint is in slight flexion to allow visualization).

LM and DP did not evidence abnormalities (Figures 2A,B). The LM flexed projection exhibited slight remodeling of the dorsoproximal aspect of the first phalanx without evidence of a loose intra-articular fragment (Figure 2C). The DMPLO projection revealed a faint radiolucent area over the lateral condyle (Figure 2D).

#### 3.2.3 Arthroscopy

Arthroscopy was performed to explore the LF MCj, to eliminate the possibility of a proximal P1 osteochondral chip fracture, to precisely assess the nature and extent of the radiological findings at MC3 and P1, and therapeutically address the pathological evidence (Figure 1, Day = 30). The patient underwent premedication with penicillin procaine (Depocilina 300 mg/mL^®^, 12 mg/Kg, IM, MSD Portugal), phenylbutazone (Phenylarthrite^®^, 2.2 mg/kg, IV, Vetoquinol, France), and detomidine (Domosedan^®^, 0.02 mg/kg, IV, Orion Corporation, Finland). The arthroscopic examination was performed under general anesthesia with the patient in dorsal recumbency. Induction was performed with ketamine (Ketamidor^®^, 2.2 mg/Kg, IV, Richter Pharma, Austria) and midazolam (Dormazolan^®^, 0.02–0-08 mg/kg, IV, Dechra, United Kingdom), and maintenance was under gas anesthesia with isoflurane (Isovet^®^, Piramal Critical Care Limited, Netherlands) and a continuous infusion of romifidine (Rominervin^®^, 0.025 mg/Kg, IV, Dechra, United Kingdom).

A standard dorsal approach was performed to the left MCj, and an 18 cm length x 4.5 mm diameter scope (KARL STORZ^®^ SE & Co. KG, Tuttlingen, Germany) was introduced into the joint.

Following mild synovectomy to enhance visualization and lesion assessment, the following lesions were identified:

(1) A linear partial thickness lesion extending distally through the axial aspect of MC3 lateral condyle (Figure 2E blue); a (2) semilunar-shaped partial thickness lesion dorsoproximal aspect of P1 (Figure 2E red); a (3) circular partial thickness lesion on the lateral side of the sagittal ridge (Figure 2F pink). With slight flexion of the joint, the main lesion was visible on the middle aspect of the lateral condyle, and this was a (4) full-thickness linear lesion of approximately 7 mm in length and covered with fibrillated fibrin (Figure 2G green). This lesion was debrided using a combination of bone curettes and a synovial resector down to the healthy subchondral bone plate at a depth of 2 mm (Figure 2H green). Immediately adjacent to this lesion, there was a similar (5) linear lesion in the first phalanx, suggesting that these lesions were correlated and that the MC lesion had led to abrasion of the adjacent P1. The latter was debrided in a similar fashion, but its depth did not extend further into the subchondral bone (Figure 2H yellow).

The time gap between the first assessment of the patient and the arthroscopic examination was based on reasons other than clinical principles, as this was an outpatient ambulatory clinic.

#### 3.2.4 Magnetic resonance image

MRI of the MCj was performed under the same general anesthesia protocol previously described at arthroscopy. A single dose of flunixin meglumine (Flunixin 3E^®^, 1.1 mg/kg, IV, Norbrook Laboratories, Ireland) was administered. The protocol included sagittal—Time weighted image (TW) 2*W and Short Tau Inversion Recovery (STIR), dorsal—T1W GRE and STIR, and transverse—T1W multiecho gradient recalled echo (GRE), T2W fast spin echo (FSE), and STIR. Two MRI exams were performed approximately 6 months apart (Figure 1, D = 135 and D = 315).

#### 3.2.5 Computed tomography scanning

Cone-beam CT scans were conducted simultaneously with each MRI session (Figure 1, D = 135 and D = 315).

#### 3.2.6 Computed tomography arthrography

A CTA was performed 6 months after the second CT scan described above to assess cartilage thickness. In brief, under standing sedation with detomidine (Domosedan^®^), 10 mL of iohexol solution (Omnipaque™ 300, GE Healthcare, United Kingdom) was injected into the MCj, and a CT scan was performed (Figure 1, D = 495). A single dose of flunixin meglumine (Flunixin 3E^®^, 1.1 mg/kg, IV, Norbrook Laboratories, Ireland) was administered.

## 4 Treatment protocol

Following clinical examination and diagnosis, the patient underwent a treatment protocol comprising two intra-articular administrations of a novel therapeutic combination of eSM-MSCs with eUC-MSC CM at the left MCj, with 15 days intervals, followed by a rehabilitation plan (Figure 1, D = 45).

The therapeutic protocol began with the patient’s sedation with detomidine (Domosedan^®^), followed by clipping of the left MCj area. The skin was aseptically prepared. The therapeutic combination was prepared and loaded into a 2-ml syringe and subsequently injected with a 20G needle, ensuring homogeneity of the solution and MSCs viability (35) through a dorsal approach into the MCj. The intra-articular therapeutic solution comprised allogenic eSM-MSCs suspended in eUC-MSC CM.

In brief, the eSM-MSCs donor was a young and healthy 6-month-old foal whose cause of death was a field accident. Collection preparation and procedures were previously described (32). After collection, the equine synovial membrane was prepared at the Laboratory of Veterinary Cell-based Therapies, ICBAS-UP. The isolation protocol of eSM-MSCs had been developed by Regenera^®^ (32). eSM-MSCs were characterized through trilineage differentiation, immunohistochemistry, and karyotype analysis. eSM-MSCs CM preparation and analysis were also performed as previously described (32). eUC-MSCs were isolated from equine UC—Wharton’s jelly—and the connective tissue surrounding UC. It was expanded to form the culture of adherent cells with fibroblastic morphology. Trilineage differentiation, immunophenotype, and bacteriological control were previously performed and described (33). CM preparation and analysis were previously described (33). This process is a patented technology owned by Regenera^®^ (33).

The therapeutic solution for intra-articular clinical application was a combination of allogenic eSM-MSCs suspended in eUC-MSC CM. Prior to the preparation of the final therapeutic combination, eSM-MSC and UC-MSC CM were produced and preserved as described above. Cryopreserved P3 eSM-MSCs batches were suspended in the treated animal’s autologous serum. Approximately 2 mL of eSM-MSCs (1×10^7^ cells) solution was suspended in UC-MSC CM, final concentration 1:1, and transferred to a perforable capped vial and preserved on ice until the moment of administration. This therapeutic combination is currently a patented technology owned by Regenera^®^ (PCT/IB2019/052006, WO2019175773). This preparation was previously described (33).

After each intra-articular administration, the limb was bandaged for 24 h, and the horse received a single dose of phenylbutazone (Phenylarthrite^®^, 2.2 mg/kg, IV, SID) to avoid any joint flare reaction after MSCs administration. The horse was closely monitored for 48 h post-treatment. After the first intra-articular administration, a 12-week rehabilitation program was initiated, beginning with 2 days of stall rest followed by 13 days of 10-min hand-walking sessions with increasing times of exercise (Table 1) (5, 33, 36–39). This set of procedures resumes the first treatment round (Figure 1, D = 135). After undergoing the prescribed program and veterinary clinical reassessments, advanced diagnostic imaging, namely MRI and CT exams, was performed to better analyze the healing of the defects and to decide the patient’s readiness to resume full work (Table 1).

After the MRI and CT scan analysis, it was decided to perform a second treatment round using the same protocol (Figure 1, D = 135). Six months later, another set of MRI and CT scans was performed (Figure 1, D = 315).

### 4.1 Treatment outcome

Clinical improvement was monitored through assessments of lameness, pain response to pressure and flexion, and fetlock swelling/inflammation. During the therapeutical administrations and throughout the course of the physical rehabilitation program, the horse did not present significant clinical signs, such as increased lameness, pain, or inflammation, requiring treatment cessation.

**Figure 3:**
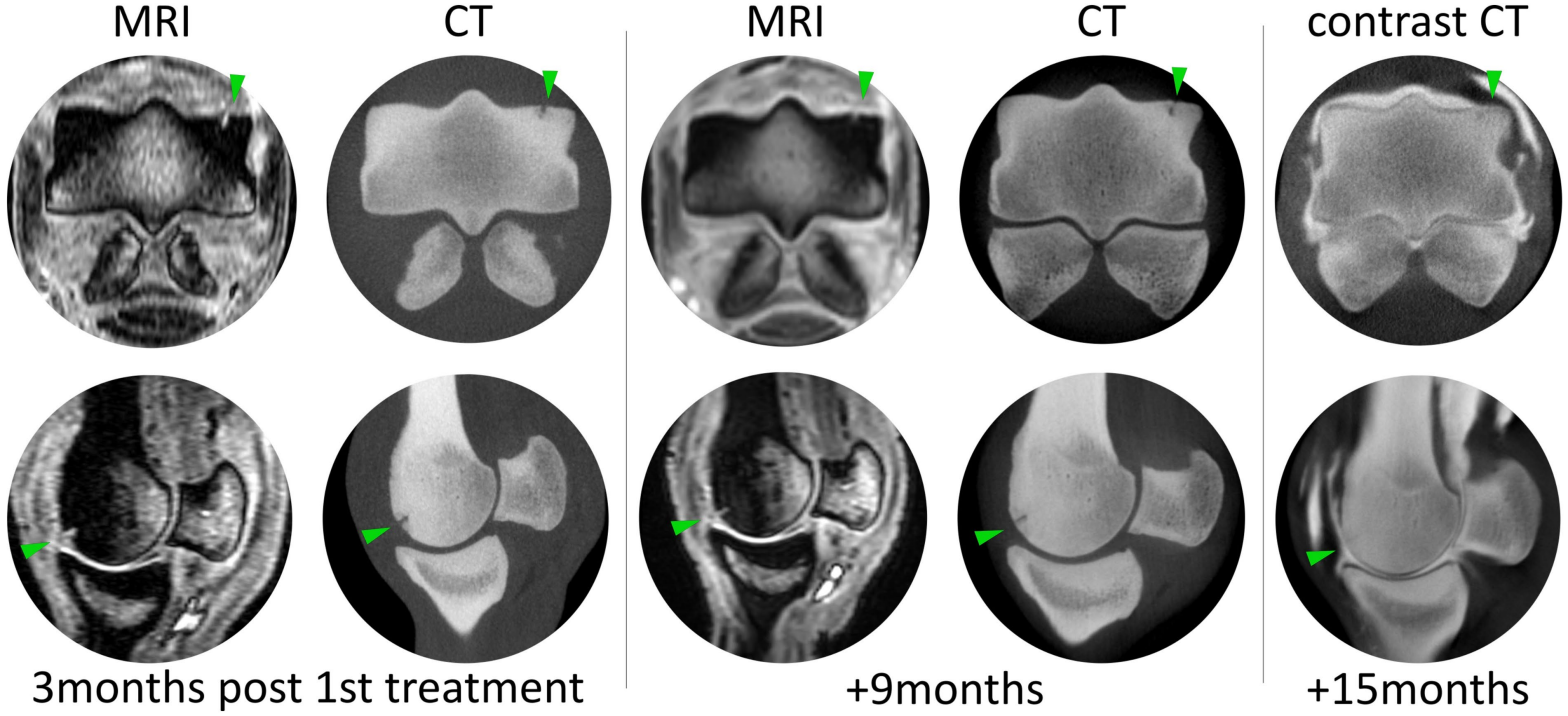
Magnetic resonance and CT imaging throughout the post-treatment period at three distinct time points. Lesion progression is evident between each time point, but the majority of the radiographic filling of the lesion occurs between 9 and 15 months post first treatment. Contrast CT allows assessment of cartilage-like tissue thickness, and although the cartilage hallow is slightly thinner than the medial condyle, there is no contrast within the defect.

Following the conclusion of the rehabilitation program, the horse exhibited residual lameness (1/5), with the absence of joint swelling and pain upon palpation. At this time, an MRI and a CT scan were performed to assess the healing of the affected tissues. It was demonstrated that the absence of P1’s partial thickness lesions was identified at x-ray and arthroscopy, but the full-thickness lesion at MC3’s lateral condyle persisted. The following findings were observed at MRI: articular and subchondral hyperintensity within the lateral condyle of the MC3 (Figure 3, “3 months post 1st treatment”); areas of mineralization (densification) within the distal epiphysis of the third metacarpal bone (larger laterally than medially); mild periarticular remodeling at the abaxial margins of the proximal epiphysis of P1; and the proximal articular margins of the proximal sesamoid bones, compatible with mild OA of the MCj. CT scans exhibited compatible changes with MRI images—articular lesion in MC3’s lateral condyle, mineralization of trabecular and subchondral bone of MC3 and P1, and signs of OA. As our treatment goal was the achievement of complete clinical recovery as well as imaging improvement of the articular cartilage and subchondral bone, a decision was made to proceed with a second round of treatments performed under the same protocol.

After the second 12-week rehabilitation plan, the lameness grade improved to 0/5, with no pain evident upon palpation, pressure, or flexion. The horse was shod with a thinner lateral branch to mitigate the impact on the lateral side of the joint and resumed normal and full work, including jumping exercises and remaining sound. The MRI performed after the conclusion of the second-round treatment demonstrated the absence of signs of joint effusion/synovitis, stabilization of OA findings, and filling of MC3’s osteochondral lesion. Regarding the lesion, it had less defined articular and subchondral hyperintensity, and the areas of mineralization within the distal epiphysis of MC3 (lateral bigger than medial) did not show progression with OA changes not developing (Figure 3, “+ 9 months”). At the second examination, a CT scan revealed more noticeable differences in OA changes than MRI. The articular surface of the lateral condyle was irregular, and a focal hypoattenuating region was seen further palmarly (Figure 3, “+ 9 months”). There was mild periarticular remodeling in both the distal aspect of MC3 and the proximal aspect of P1. These findings did not show progression between the two exams performed 6 months apart.

The horse remained in full work, and 6 months after the last MRI and CT scan, a CTA demonstrated total fulfillment of MC3’s lesion with the resumption of bone structure and the presence of a thin layer separating the bone from contrast and no contrast within the lesion confirming lesion fulfillment (healing) and partial cartilage-like tissue recovery (Figure 3, contrast CT).

Twenty months after the first treatment, the horse remains sound and has returned to full athletic performance as a young showjumper.

## 5 Discussion

Articular cartilage is a challenging tissue to regenerate (40). Assessment of the healing effect of orthobiologics can be challenging in cases of diffuse OA; the occurrence of localized lesions, such as those presented here, provides a unique opportunity to enhance our understanding of the effectiveness of regenerative treatments. This study describes a clinical case of a young horse with a traumatic lesion to the MCj, treated with an innovative therapy combining eSM-MSC and UC-MSC CM.

Using different imaging techniques and clinical follow-up, we were able to assess the safety and outcomes of the treatment. Twenty months post-treatment, the patient resumed jumping activities and competitions, exhibiting no clinical symptoms or relapses detected by diagnostic imaging.

This horse underwent two treatment rounds using the aforementioned combination therapy. Following the initial treatment round, a significant improvement in lameness score indicated a reduction in acute joint inflammation. However, the osteochondral lesion was insufficiently fulfilled, highlighting slow tissue healing. This emphasizes the need for comprehensive monitoring during the healing phase, as decreased pain may tempt an early return to exercise when tissue integrity is suboptimal, risking incomplete healing and potential recurrence (41–45). After the first treatment round, MRI and CT scan exams evidenced the absence of P1’s and MC3’s partial thickness lesions and increased mineralization in MC3’s subchondral bone, suggesting osteochondral regeneration. However, a lesion at MC3’s lateral condyle, approximately 7 mm in depth, was demonstrated. There were significant improvements in both P1 and MC3 lesions but no complete healing of MC3’s full-thickness lesion was observed.

At the second treatment round conclusion, 3 months after its beginning, the horse exhibited clinical soundness with no lameness and no radiological abnormalities. MRI and CT scans indicated the absence of lesions in P1’s cartilage, with no progression of OA and MC3’s osteochondral defect, which was notably less prominent. The absence of OA progression was considered a positive prognostic indicator, suggesting effective pathology control and prevention of further deterioration (35, 46, 47). The observed improvement in lesion fulfillment was interpreted as regenerative, reflecting a significant advantage of this treatment.

Six months later, the CTA confirmed the presence of a thin layer of articular cartilage-like tissue in MC3’s condyle. As the horse was sound, in full work, and competing, a histologic examination was not performed to distinguish between cartilage and fibrocartilage. However, the fulfillment of the lesion, the achievement of clinical soundness, and the athletic performance were considered as successful attainment of this protocol treatment.

As it was previously demonstrated, SM-MSCs can delay the progression of cartilage degeneration in osteoarthritis, relieving pain and improving joint function, and are also able to regenerate cartilage and subchondral bone (48, 49), as could be observed in this case. SM-MSCs and UC-MSCs present chondrogenic ability, high proliferative potential, and low immunogenicity (50), being able to modulate inflammatory processes related to musculoskeletal injuries (11, 40, 48, 49, 51) and regenerate osteochondral defects (49). In this case, it is suggested that the combination of SM-MSCs and UC-MSC CM may have had a positive effect on the clinical and diagnostic imaging outcome (52), enabling the patient’s return to competition.

The combined MRI and CT assessment provided valuable insight into the case’s progression, offering a thorough analysis of osteochondral fulfillment and OA development. This comprehensive evaluation provided essential information, enhancing the understanding of the procedure’s success and effectiveness (49).

The rehabilitation program may have played a crucial role in the overall treatment protocol, ensuring that the horse remained consistently active and received a continuous and progressive stimulus on its cartilage, joints, ligaments, and muscles. In contemporary equine sports medicine, it is well-established that physical rehabilitation is a pivotal component, emphasizing the importance of keeping the horse engaged in exercise (37, 38, 53, 54). The incorporation of early mobilizations is recommended as long as rehabilitation protocols are followed carefully. These mobilizations encompass weight-bearing activities, straight-line walking, and strengthening and flexibility exercises (38, 55, 56).

In addition, the importance of physical loading in chondrocyte maturation and phenotype maintenance is widely acknowledged, with diminished biomechanical loading often resulting in atrophy (57–59). Bearing this in mind, a comprehensive understanding of the impact of mechanical stimulation on MSC chondrogenic differentiation can enhance the effectiveness of MSC-based cartilage regenerative therapies, particularly in joints subjected to a mechanically demanding environment (40). Basically, the importance of controlled and purposeful physical activities during rehabilitation contributes to the overall success of the treatment, promoting optimal tissue healing and functional recovery (53).

Comparing the outcomes with the limited existing literature, in this case, the patient was sound and returned to work and competition in a reduced time frame (60–62). In an equine radius articular defect treated with ACI, the horse stood in rest for 4 weeks (vs. 2 days in our protocol) and was sound, having recovered from joint distension after 24 months (vs. 12 months and 3 months with our protocol) (60). In another report describing MSC joint capsule rupture treatment, the horse re-established its athletic performance 1 year later. Comparatively, a complex joint injury with both partial and full-thickness articular defects of a forelimb fetlock, such as the one herein described, also completely recovered after 12 months and restored athletic performance after 13 months (61). In another group of horses with stifle injury treated with bone marrow-MSCs (BM-MSCs), 43% of the horses returned to previous level work only 24 months after treatment of MSC (62).

Concerning the number of doses and cells applied in this therapeutic protocol, a total of four allogeneic doses of 1 × 10^7^ cells, all from the same donor, were performed. None of them yielded any local or systemic adverse effects. In fact, the number of applied cells is consensual among equine MSCs clinical studies (35, 63, 64). Notably, doses of 1 × 10^7^ cells demonstrated superior clinical improvements compared to higher injected doses (49, 51). The clinical intra-articular application of MSCs is an easy, minimally invasive, and reasonably safe procedure, with no reported serious adverse events (48, 49, 51, 65). These findings emphasize the effectiveness, safety, and easy-to-use characteristics of the applied MSC therapy in this particular case.

Since there are no well-defined protocols for applying MSC-based therapies in horses, the administration scheme herein applied was decided based solely on the patient’s clinical outcome. Research involving equine and humans has demonstrated the advantages of increasing the number of intra-articular MSC therapeutic applications (2 administrations and 3 administrations per year) with no clinically relevant side effects (66–68). Therefore, repeated intra-articular administrations are documented and advisable, and in this case, it is believed that it supported treatment effectiveness. Potential limitations regarding repeated intra-articular injections concern the fact they might induce both primary and secondary humoral immune responses. However, MSCs preconditioned with pro-inflammatory cytokines demonstrated an enhanced immunomodulatory capacity. This preconditioning potentially enabled these cells to more efficiently manage inflammation, reducing a primary humoral response upon initial administration (25).

To sum up, the intra-articular administration of this novel therapeutic formulation, combining eSM-MSCs and eUC-MCS CM, demonstrated a successful efficacy in treating both partial and full-thickness cartilage defects in this equine patient. Subsequent assessments revealed notable advancements in clinical and imaging parameters. Remarkably, the patient exhibited complete clinical recovery, restoring athletic activity, and even surpassing the pre-treatment sportive level. Limitations of the current study relate to its observational nature, wherein the diagnostic exams used during the therapeutical follow-up were not employed at the initial diagnosis stage, and, also, after CTA, no arthroscopy and biopsy were performed to guarantee the real nature of the cartilage tissue. However, the horse has completed full function, being in competition, and for that reason, no more invasive exams were performed. Additionally, the absence of a control group further restricts the ability to draw definitive conclusions. The potential benefit of the rehabilitation program should also be considered.

Considering this, further investigations involving a more extensive cohort of equine patients with comparable lesions undergoing meticulous follow-up assessments under standardized conditions are imperative to definitively ascertain the effectiveness of this innovative and auspicious therapeutic protocol.

## 6 Conclusion

Articular cartilage defects in equine joints, particularly the MCj, can lead to significant lameness and inevitably trigger OA development. However, with a precise diagnosis, a positive outcome might be achieved with regenerative treatments, namely with this innovative treatment. The combination of eSM-MSC and UC-MSC CM was demonstrated to be safe and effective, as no adverse signs were reported, and advanced imaging evidenced fulfillment of the osteochondral lesion, absence of the other lesions previously identified at arthroscopy, and OA stabilization. In addition, and no less important, clinically, after a 20-month follow-up period, the equine returned to and progressed in its athletic career without any signs of lameness or clinical relapse, no swelling or pain of the injured area, now competing at a higher level.

## Data availability statement

The original contributions presented in the study are included in the article/supplementary material, further inquiries can be directed to the corresponding author.

## Ethics statement

The animal studies were approved by ORBEA – Órgão Responsável em Bem-Estar Animal (ICBAS). The studies were conducted in accordance with the local legislation and institutional requirements. Written informed consent was obtained from the owners for the participation of their animals in this study.

## Author contributions

IR: Conceptualization, Data curation, Formal analysis, Investigation, Methodology, Writing – original draft. BL: Formal analysis, Investigation, Methodology, Writing – original draft. PS: Conceptualization, Data curation, Formal analysis, Investigation, Writing – original draft. AS: Conceptualization, Formal analysis, Investigation, Methodology, Visualization, Writing – original draft. ARê: Formal analysis, Investigation, Methodology, Writing – original draft. AC: Conceptualization, Investigation, Methodology, Writing – original draft. IB: Investigation, Methodology, Writing – original draft. ARo: Investigation, Methodology, Validation, Writing – original draft. JP: Formal Analysis, Investigation, Methodology, Validation, Writing – original draft. CM: Data curation, Investigation, Methodology, Supervision, Writing – review & editing. JS: Supervision, Writing – review & editing. LL: Formal analysis, Investigation, Methodology, Supervision, Validation, Writing – review & editing. LA: Investigation, Methodology, Supervision, Validation, Writing – review & editing. RA: Data curation, Investigation, Validation, Writing – original draft, Writing – review & editing. AM: Conceptualization, Data curation, Formal analysis, Funding acquisition, Investigation, Methodology, Project administration, Resources, Supervision, Validation, Writing – original draft, Writing – review & editing.

## Glossary

AAEP: American Association of Equine Practitioners
ACI: Autologous chondrocyte implantation
BM-MSCs: Bone Marrow mesenchymal stromal/stem cells
CM: Conditioned Medium
CT: Computed Tomography
CTA: Computed Tomography arthrography
D: Day
DLPMO: Oblique Dorsolateral-palmaromedial
DMPLO: Oblique dorsomedial-palmarolateral
DP: Dorso palmar
ECM: Extracellular matrix
eSM-MSC: Equine synovial membrane mesenchymal stem/stromal cell
eUC-MSC CM: Equine Umbilical cord mesenchymal stem/stromal cell conditioned medium
Flex: flexed
FSE: Fast scan echo
G: Gauge
ILGRE: Gradient recalled echo
IA: Intra-articular
ICBAS-UP: Instituto de Ciências Biomédicas Abel Salazar – Universidade do Porto
IL: Interleukins
IV: Endovenous
Kg: Kilogram
Kv: Kilovolt
LF: Left forelimb
LM: Lateromedial
mA: milliampere
MC3: Third Metacarpus
MCj: Metacarpophalangeal joint
mg: milligrams
min: minutes
mL: milliliter
MRI: Magnetic Resonance Image
MSCs: Mesenchymal Stem/Stromal Cell
OA: Osteoarthritis
ORBEA: Organismo Responsável pelo Bem-estar Animal
P1: First phalanx
PLDMO: Oblique palmarolateral-dorsalmedial
PMDLO: Oblique palmaromedialdorsolateral
SID: Once a day
SM: Synovial membrane
SM-MSC: Synovial Membrane Mesenchymal Stem/Stromal Cell
STIR: Short Tau Inversion Recovery
TW: Time weighted image
UC: Umbilical cord
UC-MSC MC: Umbilical cord mesenchymal stem/stromal cell conditioned medium
Vet-check: Veterinary check-up
W: weight
WJ: Wharton Jelly
X-ray: Radiograph
